# Multidimensional Mutational Profiling of the Indian HNSCC Sub-Population Provides IRAK1, a Novel Driver Gene and Potential Druggable Target

**DOI:** 10.3389/fonc.2021.723162

**Published:** 2021-11-02

**Authors:** Sagar Sanjiv Desai, Raksha Rao K, Anika Jain, Pushpinder Singh Bawa, Priyatam Dutta, Gaurav Atre, Anand Subhash, Vishal U. S. Rao, Suvratha J, Subhashini Srinivasan, Bibha Choudhary

**Affiliations:** ^1^ Department of Biotechnology and Bioinformatics, Institute of Bioinformatics and Applied Biotechnology, Bangalore, India; ^2^ Graduate Student Registered Under Manipal Academy of Higher Education, Manipal, India; ^3^ Department of Biotechnology, School of Biosciences and Technology, Vellore Institute of Technology, Vellore Campus, Katpadi, Vellore, India; ^4^ Healthcare Global Enterprises Ltd, Cancer Centre, Bangalore, India

**Keywords:** HNSCC, IRAK1, survival, driver gene, LASSO, ROC, whole exome sequencing

## Abstract

Head and neck squamous cell carcinomas (HNSCC) include heterogeneous group of tumors, classified according to their anatomical site. It is the sixth most prevalent cancer globally. Among South Asian countries, India accounts for 40% of HNC malignancies with significant morbidity and mortality. In the present study, we have performed exome sequencing and analysis of 51 Head and Neck squamous cell carcinoma samples. Besides known mutations in the oncogenes and tumour suppressors, we have identified novel gene signatures differentiating buccal, alveolar, and tongue cancers. Around 50% of the patients showed mutation in tumour suppressor genes TP53 and TP63. Apart from the known mutations, we report novel mutations in the genes AKT1, SPECC1, and LRP1B, which are linked with tumour progression and patient survival. A highly curated process was developed to identify survival signatures. 36 survival-related genes were identified based on the correlation of functional impact of variants identified using exome-seq with gene expression from transcriptome data (GEPIA database) and survival. An independent LASSO regression analysis was also performed. Survival signatures common to both the methods led to identification of 4 dead and 3 alive gene signatures, the accuracy of which was confirmed by performing a ROC analysis (AUC=0.79 and 0.91, respectively). Also, machine learning-based driver gene prediction tool resulted in the identification of IRAK1 as the driver (p-value = 9.7 e-08) and also as an actionable mutation. Modelling of the IRAK1 mutation showed a decrease in its binding to known IRAK1 inhibitors.

## Introduction

Head and Neck cancer (HNC) is a heterogeneous disease that encompasses tumors of majorly three regions, oral cavity, oropharynx, and larynx, and together they account for more than 660000 new cases and over 320000 deaths worldwide wide, while India has contributed to 36%, 20.9% and 18.8% of the total cases of each type respectively, in 2020 (https://gco.iarc.fr) (1). More than 90% of the HNCs are diagnosed as squamous cell carcinomas (HNSCC). The common risk factors worldwide are smoking tobacco, alcohol consumption, improper diet, whereas chewing areca nuts, chewing tobacco, smoking bidis, etc., are rampant in India (1, 2). Over the past 3-4 decades, various treatment regimens like surgery, adjuvant chemotherapy, radiation therapy, immunotherapy, etc. have been employed, yet only 50 % improvement in survival rates have been achieved for HNSCC (3–5).

Several studies have reported drivers of Head and Neck cancer oncogenesis. The drivers can be broadly classified into tumor suppressors and oncogenes. Alterations in oncogene families like *ras* family of genes, *myc* family and EGFR family have been implicated in oral and head and neck cancers. High frequency of mutations in *HRAS*, copy number alterations and aberrant expression levels in *KRAS, NRAS, MYC* and *EGFR* have been reported in relation with development of many squamous cell carcinomas. Driver Genes like *CCND1, MAPK* family and *PIK3CA* are involved in the progression of HNSCC (4, 6, 7). Early stages of head and neck cancers have been associated with inactivated *CDKN2A* and *TP53*, loss of function copy alterations is associated with aggressive cancers. HPV+ve HNSCC cancers are characterized by frequent mutations and chromosomal deletions in tumor suppressors like *PTEN*, E-cadherins and *RB1 (* 4, 6, 8). One of the first steps of oncogenesis involves the evasion of immune system. In HNSCC, *IRAK1* overexpression is associated with tumor progression and low survival (9). *IRAK1* is a kinase, activated downstream of TLRs and is activated upon radiation therapy in HNSCC (10, 11)

Several models have been proposed as predictive biomarkers for the prognosis of HNSCC patients. A recent study reported a 6 gene signature for predicting survival in patients using random forest sampling and Cox regression analysis. Exome seq analysis has led to the identification of SNPs in the genes, which can be used as independent prognostic markers (12, 13). Oncogenic driver mutations in genes commonly associated with HNSCC, like *P53, PI3-AKT* pathway, *HRAS, CCND1* and others, have been associated with poor survival and have been identified as important factors for outcome predictions in HNSCC cohorts (4, 8). Accumulation of structural variants such as Copy number variation (CNV), Loss of Heterozygosity (LOH) in oncogenes, and tumor suppressors like *c-MYC, EGFR, CDKN2A*, respectively, have been associated with recurrence of squamous cell carcinomas, and poor prognosis and outcome predictions have been linked to rapid occurrence rates of SCNAs across tumor genomes (4, 14, 15). The genomic analyses of HNSCC from (110 patients) Indian population led to the identification of 5 new frequently mutated (10-22% of the patients) genes associated with OSCC-GB, namely, *USP9X, MLL4, ARID2, UNC13C* and *TRPM3* (16).

In this study, we have performed exome sequencing of 51 individuals from diverse anatomical sites and correlated the clinical phenotype to the genotype. We report distinct anatomical site-specific signatures and heterogeneity within each group. We have also identified novel driver mutations using Oncodrivclustl. We identified alive and dead signatures using two different approaches. The first approach was based on the correlation of functional impact of variants using exome-seq with gene expression using transcriptome data (GEPIA database) and the second was LASSO regression. The signatures were validated using a receiver operating characteristics (ROC) model. We have identified 2 missense mutations in IRAK1, one of which causes structural changes in the protein, possibly leading to change in its activity.

## Methodology

### Subjects for the Study

We obtained 51 FFPE (Formalin Fixed Paraffin Embedded) samples diagnosed with oral cancer at the Healthcare Global Enterprises Ltd, Bengaluru, Karnataka, India. The protocol was approved by the institutional review board of HCG and Institute of Bioinformatics and Applied Biotechnology. The clinical details of every patient are mentioned in **Table 1**. Informed consent was obtained from all the participants.

**Table 1:** A table summarizing clinical data of the samples in the study.

Characteristics		Number of Samples
Age (years)	<= 55	25
	> 55	21
Survival Status	Alive	23
	Dead	14
Gender	Female	15
	Male	31
Tumor Stage	T1	9
	T2	5
	T3	5
	T4	11
Habits	Smoking	18
	Alcohol	8
	Quit	3
	None	15

### Exome Library Preparation

To prepare libraries for Whole Exome Sequencing, 100ng-1µg of genomic DNA was sheared with the Covaris S220 (Covaris, Woburn, MA, USA), followed by end-repair, 3’ end Adenylation and ligation with paired-end adaptors. Post ligation, 15µl of the purified libraries were PCR amplified, all the above steps were performed using the Agilent SureSelect^XT^ kit and every step was followed by DNA purification on a magnetic stand using AMPure XP Reagent beads (Beckman Coulter Genomics, Danvers, MA, USA). Afterward, size (approx 225-275 bp) and quantity (>800ng) were verified employing the Agilent Tapestation 2200 system followed by hybridization and probe capture using Exome SureSelect Human All Exon V6+UTR probes (Agilent). Dynabeads MyOne Streptavidin T1 magnetic beads (Thermofisher). Finally, Captured Libraries were amplified with 12 cycles of PCR using indexing primers containing 8-bp indices, followed by an amplification using AMPure XP beads (Beckman Coulter). Final libraries were checked for quality (each fragment size approx. 300-400 bp) and quantity using Agilent Tapestation 2200 system.

### Whole Exome Sequencing and Analysis

The libraries were multiplexed and pooled followed by a 100-bp paired-end sequencing with ~100X exome coverage depth per sample (Approx. 60-90 mb exome size) on the Illumina HiSeq 2500 platform. The exome sequencing raw data is available at https://www.ncbi.nlm.nih.gov/sra/PRJNA740146. Filtered high exome-sequencing reads generated on HiSeq 2500 were analyzed using FastQC for quality checking (https://www.bioinformatics.babraham.ac.uk/projects/fastqc/). Bowtie2 (17) aligner was used for alignment and mapping of reads against the hg38 version of the human genome, with default parameter settings. SAMtools (18) was used for conversion of SAM files to BAM files. PCR duplicates were removed using Picard tools (http://broadinstitute.github.io/picard/). Variant calling was performed using the best practices Mutect2 module of GATK (Genome Analysis Toolkit, Broad Institute) including local realignment around insertions/deletions and base-quality score recalibration (19). Duplicate removed alignment files were subjected to another format Variant calling using pileup utilities from BCFtools (18). Variants common to pileup approach and GATK process, and the ones being spanned by more than 3 reads were annotated using the SnpEff and SnpSift tools (20, 21). Only the variants not present in the 1000G database were considered for further analysis (22).

### Obtaining Mutation Profiles

All the vcf files were primarily processed using shell scripts. Mutation frequency of genes known to be implicated in Head and Neck cancer was depicted using a waterfall plot from the R package GenVisR (23). The samples were grouped into categories based on age (0-40 yrs.; 40-50 yrs.; 50-60 yrs.; 60-70 yrs.; 70-80 yrs.), habits (alcohol; tobacco; all; none; quit), stage of tumor at the time of biopsy (early; advanced; recurrent) and site of tumor (alveolar; buccal mucosa; tongue). Total number of mutations, number of high impact mutations and number of protein coding mutations (high impact + missense variants), per sample, from each category were obtained. Mutation signatures were obtained using the SomaticSignatures package of R (24). CNV analysis was performed using CNVkit (25).

### Driver Gene Analysis

To predict the driver genes, vcf files of all the samples were merged into a single file using the BCFtools toolkit which included all the mutations present in each and every sample. This merged vcf file was given as an input to OncodriveCLUSTL for driver gene analysis (24, 26). The resulting genes were filtered based on their p-value significance and frequency of mutation. Further we checked for the effect of mutations being harbored by these genes on patient survival and it was seen that none of the mutations showed a significant difference in survival. We also checked for an association between expression patterns of these genes and patient survival from the GEPIA database (24, 26, 27) and the genes showing a significant correlation were further shortlisted followed by generating a lolliplots of the mutations present in the shortlisted genes using G3Viz (28).

### IRAK1 Structure Modeling and Validation

The IRAK1 protein (identified as one of the driver genes) structure and its two mutants S532L and F196S were modeled using the 712-residue sequence from UniProt (UniProt ID: P51617) on the Robetta web server (29). The structure obtained is further energy minimized on Swiss-PdbViewer using a GROMOS 43B1 force field to repair distorted geometries (30). The energy minimized protein structure of IRAK1 was further validated using the SAVES webserver (https://saves.mbi.ucla.edu/) which employs tools such as ERRAT (30, 31), PROVE (32), PROCHECK (32, 33), WHATCHECK (34) and VERIFY 3D (35). Site Directed Mutator (SDM) and I-Mutant 2.0 (36, 37). Webservers were used to predict the effect of mutation on the stability of the protein structure and the Gibbs free energy change(ddG). Autodock 4.0 was used to blind dock the ligand JH-X-119-01 (a selective inhibitor for IRAK1) (36–38) with the wild type IRAK1 structure as well as the S532L and F196S mutant structures to study the change in ligand binding upon mutation. The active site of *IRAK1* (Serine/Threonine Protein Kinase) corresponds to residues 336-348. The binding site of *IRAK1*(Protein Kinase, ATP Binding Site) corresponds to residues 218-239 as indicated by InterPro. LigPlot+ software (36–39) was used to visualize the ligand interactions. To understand the change in interaction between the residues in the protein structure upon mutation, Residue Interaction Analysis was performed. RING web server was used to generate a Residue Interaction Network (RIN) wherein the nodes represent the residues and the arcs represent the physico-chemical interactions (40). The network thus generated is visualized in Cytoscape to study the change in interactions of residue 196 and 532 upon mutation (41).

### Survival Models

Mutation profiles and the associated clinical data of 178 Oral cancer patients from Indian Cohort were downloaded from the ICGC database. For survival analysis, ICGC and inhouse data were clubbed. To associate demographic and clinical parameters with survival time and to assess the effect of variants on it, construction of Kaplan-Meyer plots and hazard ratio (HR with 95% CI) calculations were performed by employing univariate and multivariate Cox analysis using the survminer (https://github.com/kassambara/survminer) and survival (42) packages of R. From the inhouse data, a total of 36 genes were shortlisted based on an initial scrutiny, 19 fitting the dead signature criteria and 17 fitting the alive signature criteria, 6 genes were shortlisted based on the significant association of their mutation profiles with alive and dead samples (Chi-square analysis performed in R) and an association of expression patterns with survival analysis from GEPIA database (Gene Expression Profiling Interactive Analysis). Further functional annotations were performed using information databases such as UCSC and GeneCards.

### Lasso Regression Model

LASSO stands for Least Absolute Shrinkage and Selection Operator. It is a linear form of regularization technique (to minimize the error because of overfitting of data while constructing a model). As the name suggests, it uses a “shrinkage/penalty term(lambda)” in its regression equation to be able to predict with accuracy and precision.

Mathematical equation:


$${\sum_{i=1}^{n}\left(y_{i}-\sum_{j}x_{ij}\;\beta_{j}\right)}^{2}+\lambda \sum_{j=1}^{p}\left|\beta_{j}\right|$$

where, λ, the penalty factor (or the shrinkage parameter) and β are the coefficients related to p features (43). The significant genes were found by computing the coefficients of Lasso regression of the cox survival data. This was achieved by using the “sksurv” or “scikit-survival” module of python, present as a part of “scikit-learn”.

The equation the module sksurv.linear_model.CoxnetSurvivalAnalysis uses is as follows:-


$$\arg_{\beta }^{\max}\log PL(\beta )-\alpha \left(\sum_{j=1}^{p}\left|\beta_{j}\right|+\frac{1-r}{2}\sum_{j=1}^{p}\beta_{j}^{2}\right)$$

This equation represents the “elastic net regression”. Here *α* is the same as γ which is mentioned in the Lasso regression introduction equation. By giving a l1_ratio value of 1.0 we are eliminating the Ridge regression term (second term) and only keeping the Lasso regression term. Steps followed for each dataset; 1. The dataset was processed by “CoxnetSurvivalAnalysis” with a subset of 100 random alpha values, a l1_ratio of 1.0(complete Lasso regression), alpha_min_ratio is set to auto depending on the no. of samples and no. of features. This gives us a subset of coefficients corresponding to that particular alpha value. To obtain the best alpha value by a 5-fold cross validation, we use the following modules and their utility classes a. Sklearn.pipeline.make_pipeline b. sklearn.preprocessing.StandardScaler c. sklearn.model_selection.KFold d. sklearn.model_selection.GridSearchCV. After the best alpha value for the dataset is obtained, we obtain the coefficients for all the features pertaining to that alpha value.

### Statistical Analysis

Chi-square analysis was performed to determine the significance in difference between number of alive and dead patients per gene. Survival time was defined as days from the initial diagnosis to the death or the last follow-up. The hazard ratio and their 95% confidence intervals (95% CI) for the associations of clinical variables with survival time were calculated by univariate Cox proportional hazard analysis using the survival and survminer packages of R (42). The associations between SNPs and patient survival (for the additive 4 dead and 3 alive gene models) were analyzed by multivariate Cox regression models. The difference in survival time between different patients based on genotypes of the dead and alive signature genes, using an additive model, was assessed by Kaplan-Meier curves, the significance of the influence of the clinical parameters and the additive gene models on patient survival was determined using log-rank test. Receiver Operating characteristic (ROC) curve was constructed and the area under the curve (AUC) was used to assess the performance of the model. The results were considered significant if the p-value was less than 0.05.

## Results

### Identification of Mutation Burden and Signatures Associated With the Age and Stage of HNC Tumor

**Figure 1:**
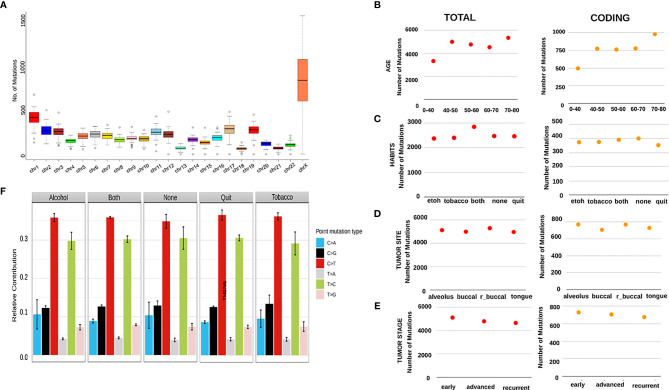
**(A)** A boxplot showing an aggregate of number of mutations in every sample per Mb of across all the chromosomes. It can be seen that chrX shows the highest number of mutations with the highest number of variations between the samples, with the next highest being chr1. X-axis shows the chromosome numbers and Y-axis depicts the number of mutations per Mb. **(B–E)** The scatter plots here show varying patterns of average number of all mutations and average number of coding mutations per category. **(A)** shows the average numbers across age groups, **(C)** shows the average numbers across patient habits, **(D)** shows average numbers across tumor sites and **(E)** shows the numbers across stages of tumors. **(F)** This is a bar graph showing the relative contribution of 6 mutational signatures in patients categorized by habits.

Variant analysis was performed for 50 Head and Neck Squamous Cell Carcinoma (HNSCC) tumor samples using exome-seq. The depth covered per sample was approximately 100X with an average of 61 million reads (**Supplementary Table 1**). To identify if there was a chromosome bias for mutations, we analyzed the mutations from all the samples. The number and density of mutation was highest on ChrX (average 794 variants) followed by Chr1 (average 400 variants), while chr18 had the least number of variants (average 60 variants) (**Figure 1A**).

To check whether the number of mutations correlate with disease progression, habit and age of the individual, we catalogued the number of mutations per individual, habit and age. On comparison across different age groups, we observed the lowest number of total variants (approx. 3000/sample) in patients aged < 40 years, while the number was as high as 5000 variants/sample in patients belonging 70-80 years of age (**Figure 1B**). Patients consuming alcohol or tobacco had relatively a smaller number of variants (approx. 2500/sample) as compared to the patients consuming both (approx. 3000/sample) and the ones having quit these habits, surprisingly, showed the least number of mutations (**Figure 1C**). Recurrent tumor samples obtained from the buccal mucosa and alveolar sockets harbored the maximum number of variants (approx. 5000/sample) as compared to tumors of other sites in the oral cavity (**Figure 1D**). Interestingly, a stage-wise distribution showed that tumors at the earliest stage have the highest number of mutations, followed by a gentle decline in the number of variants in the case of advanced and recurrent stages (**Figure 1E**). We further investigated mutational signatures mutational burden/Mb in tumors from patients with different habits and mild distinctions were observed. C>T followed by T>C mutations were seen in high abundance across all the habits with the highest being in the quit category, while the patients with alcohol consumption showed a relatively higher C>A signature and the ones with tobacco consumption had higher levels of C>G mutation as compared to other habit categories (**Figure 1F**). Patients consuming both alcohol and Tobacco displayed the maximum tumor mutational burden (TMB) and yet again, the samples having quit these habits show the lowest TMB (**Figure 3A**).

### Unique Signature in Cell Cycle, Apoptotic and Wnt Signaling Pathway Segregate Tumors of the Buccal Cavity From Tongue and Alveolus

**Figure 2:**
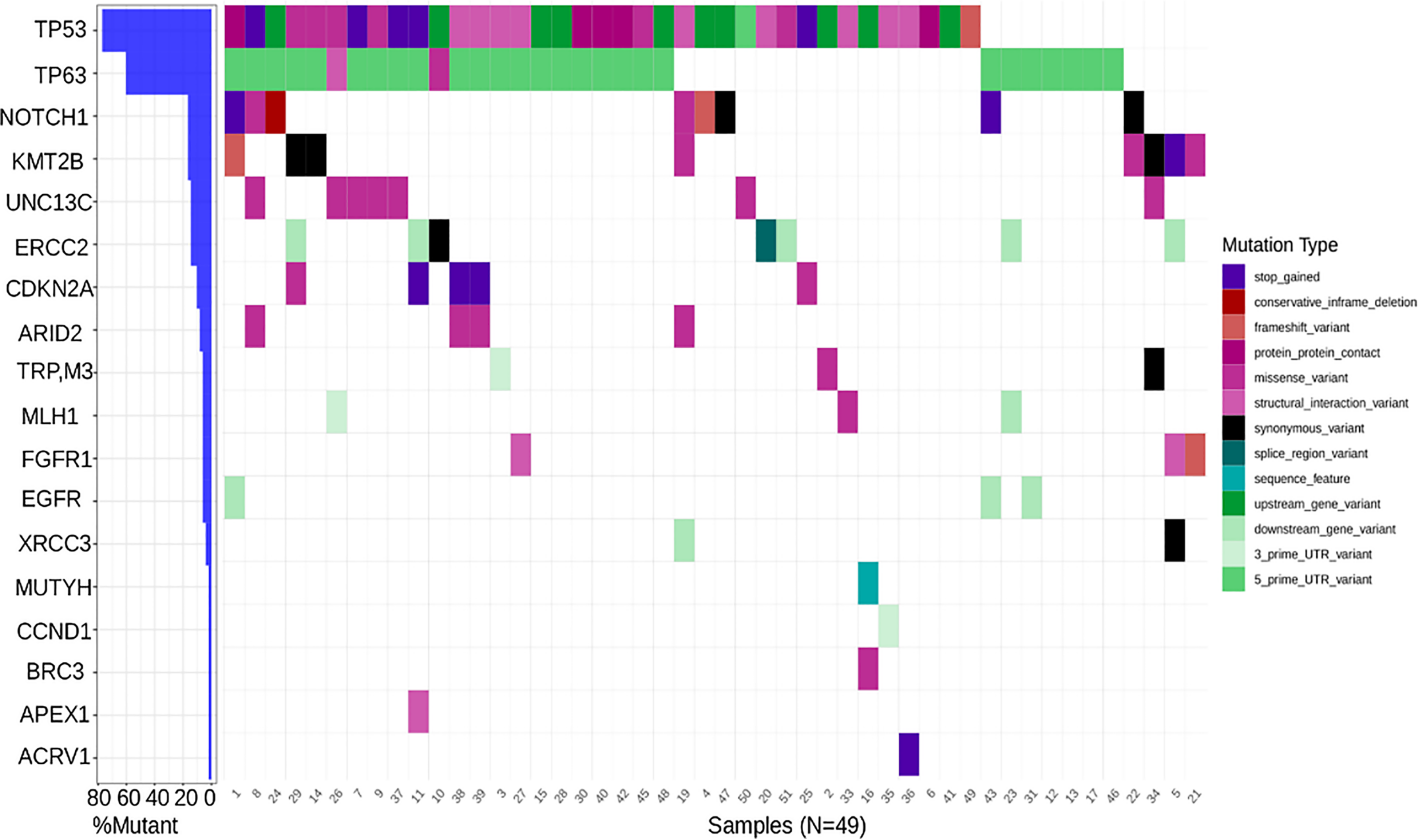
Waterfall plot depicting mutation frequency of genes known to be implicated in HNSCC across patients with the highest frequency being that of TP53 and the lowest being that of ACRV1 along with a varying frequency across tumor suppressors, cell cycle genes, DNA damage and repair genes etc.

A set of genes known to harbor somatic mutations and classified as driver genes in HNSCC were identified and a waterfall plot generated **(** **Figure 2**). As expected, *TP53*, a known tumor suppressor, and *TP63* had the highest frequency of mutation (greater than 60%) in the HNSC cohort. Transcription factors like *NOTCH1* (16%) and *KMT2B* (16%), *UNC13C* (14%), another tumor suppressor, *ERCC2* (14%) involved in nucleotide excision repair pathway, were recurrently mutated, whereas genes like *CDKN2A, MLH1, FGFR1, EGFR*, etc. had a less than 10% mutation frequency. Interestingly, *CCND1*, known to be associated with HNSCC due to copy number alterations, and *APEX1* which is known to be associated with a high risk of HNC showed negligible mutation frequency (**Figure 2**). Notably, among variations in the coding region, missense variants, stop gained variants, and structural interaction variants showed the highest frequency (**Figure 2**).

We categorized 19 patients based on anatomical sites, alveolar, buccal mucosa, and tongue and constructed waterfall plot. The genes uniquely mutated in each category were from a known list of proteins belonging to various categories namely, tumor suppressors, WNT signaling, Cell cycle, Apoptosis, EMT, Replication, etc. Tumor site data was available for only 19 of the patients. Tumour site data was available for only 19 of the patients. It was seen that *APC4*, a gene involved in Cell cycle progression, harboring a structural interaction variant showed the highest frequency being present in 18 out of 19 patients across all the tumor sites. 50-60% of the samples showed mutations in genes involved in Apoptosis, like CASP10, ATF4, PARP1, and TNFSF10, most of which were either missense or structural interaction variants, respectively. The alveolar tumours were characterized by mutations in cell cycle genes, PRKCG, RBL2, RFC2, WEE1, MCM2, CDKN2A, SMAD4, MCM3, and PRKDC proto-oncogenes like HRAS, NRAS, LMNB2. Mutational signatures in the patients with buccal mucosa category belong to the Apoptosis pathway (*MAPK10, CTSW, DAXX, ATM, HTRA2, BIRC3, CASP6*) and WNT signaling pathway (*WISP1, NOTUM, NFATC1, ROR2, CTNNB1, CACYBP*) and EMT pathway related genes, namely*, RNASEH2B, DAB2IP, MMP9* and *SNAI* were observed. Interestingly, the mutation in transcription factor *TFDP1* involved in the cell cycle was observed only in 2 samples both of them had recurrence. The least number of genes with mutational signatures were obtained in tongue cancer. Tongue cancer was characterized by unique mutations in the cell cycle genes MCM4, RNASEH2A, HDAC1, CCNB1, WNT signalling pathway LRP5, NFATC4, and WNT11, and TP53 tumour suppressor gene (**Supplementary Figure S1**). We also observed patient specific mutation within each category depicting heterogeneity in each of the samples.

### Combination of Radiotherapy, Chemotherapy, and Surgery Is Associated With Worse Patient Survival Compared to Surgery and Chemotherapy or Surgery and Radiotherapy

**Figure 3:**
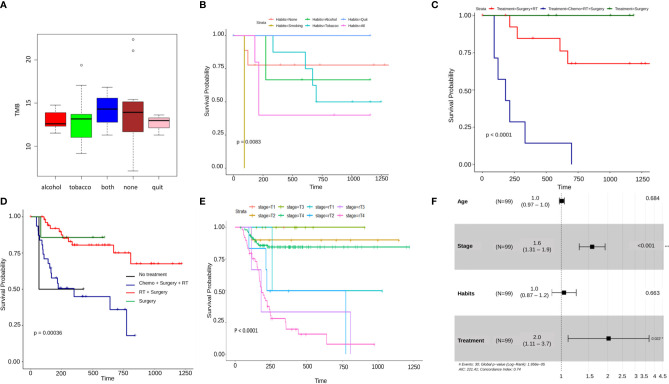
**(A)** A boxplot of average tumor mutation burden of patients categorized by habits. Patients having both the habits of alcohol consumption and tobacco usage, show the highest amount of average tmb, lowest is seen to be in the patients with alcohol consumption and those who have quit either of the habits. Surprisingly, it is seen to be high in the patients having none of the habits. **(B–E)** Kaplam-Meier plots showing significant differential survival probabilities between different clinical categories. **(B)** This plot shows a significantly lower survival in patients with the habit of smoking as compared to any other category. **(C)** This Kaplan-Meier plot depicts a significantly lower survival probability in patients with a combined treatment regimen of chemotherapy, radiation therapy and Surgery as against any other individual or combined treatment type. This pattern gets reflected again in **(D)** where the data from ICGC Indian oral cancer cohort has been combined with in-house data and finally, **(E)** shows A differential survival plot of patients from ICGC and in-house data showing a significantly lower survival probability in patients with recurrent tumor stages as against their non-recurrent counterparts. **(F)** A cox-proportional hazard ratios forest plot, depicting the fact that there is a significant difference between various categories tumor stage and treatment regimens.

Differential survival within different clinical properties was illustrated using Kaplan-Meier plots. Within the habit’s category, significantly (p=0.0083 < 0.01) low survival was observed for patients who consumed alcohol and tobacco, compared to patients having neither and the ones who have quit (**Figure 3B**). Among various forms of treatment, patients having undergone only Surgery and the ones with Surgery + Radiotherapy had a significantly higher probability of survival as compared to the patients having been administered with all three, Chemotherapy + Surgery + Radiotherapy (p<0.0001) (**Figure 3C**). For comparison, Oral cancer whole exome data of the Indian Cohort consisting of 178 samples, was downloaded from the ICGC database along with the clinical parameters of the patients. On clubbing the in-house and ICGC survival data, it was observed that patients that underwent surgery + radiation therapy showed significantly better survival (p=0.00036) as compared to the ones with only surgery and surgery + chemotherapy + radiation. In conclusion, the samples that were given surgery + chemotherapy + surgery showed the lowest probability of survival in both scenarios. As expected, the patients with no treatment administered exhibited the least survival probability **(** **Figure 3D** **)**. The tumor stage of patients along with recurrence was also analyzed and it was seen that patient showing recurrence at any stage of the tumor displayed a significantly lower probability of survival (p<0.0001) with the recurrent T4 stage showing the lowest of survival as compared to the patients with stages without recurrence. A similar trend was seen in the inhouse data but the result was only mildly significant (p-value=0.07) (**Figure 3E**, **Supplementary Figure S2A**). A multivariate Cox proportional hazards analysis of the clinical parameters revealed that the tumor stage and treatment (Hazard Ratio 1.6 and 2.0 respectively) significantly (p-value < 0.001) influence the patients’ risk of death (**Figure 3F**).

### Identification of IRAK1 and UMODl1 as Driver Genes and Potential Therapeutic Targets

**Figure 4:**
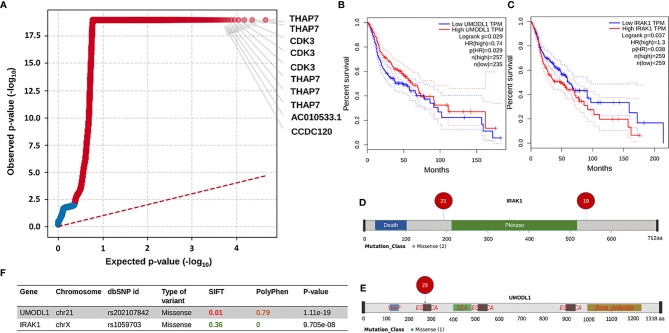
This entire panel of figures represents results from driver genes prediction and the subsequent analyses performed. **(A)** This is a quantile plot generated by oncodriveclutstl that depicts significant driver genes predicted as per frequency of mutations. This plot shows THAP7 and CDK3 as the most significant driver genes. **(B, C)** Show expression-based survival plots of two more driver genes, UMODL1 and IRAK1, respectively, from the HNSCC dataset in GEPIA2 database. Both have a significant difference in survival probability between high and low expression categories. **(F)** Table showing the details of the UMODL1 and IRAK1 mutations. It can be seen that the missense mutations in both of them have been previously reported, are deleterious and probably damaging based on the SIFT and PolyPhen values. The p-value in the last column is the significance measure from oncodriveclustl prediction. **(D, E)** Are lollipop plots depicting the amino acid position and the number of patients of the missense mutations in UMODL1 and IRAK1 linear protein structures respectively.

After getting a general idea of the mutation spectrum and survival trends associated with clinical parameters, we checked for oncogenic driver genes using OncodriveCLUSTL. Among the significant genes obtained, top two genes *THAP7* and *CDK3* (p-value=1.11e^-19,^ had mutations present in almost all the samples) (**Figure 4A**). *THAP7* harbored a missense mutation which was present in 48 out of 51 samples whereas *CDK3* had a downstream gene variant being a part of *CDK3-TEN1* fusion present in 42 samples. Further, in order to screen the other genes from the output, we started by referring to the GEPIA database. From the significant list of genes, we checked for the ones displaying a significant difference in survival from the HNSCC transcriptome dataset in GEPIA and we obtained two driver genes that had a significantly high frequency of mutations across all the patients. The first was *IRAK1*, with two missense mutations, present in 21 and 19 patients respectively (**Figure 4D**), on either side of its kinase domain with a p-value for its mutation cluster being 9.70e^-08^ (**Figure 4F**) and significant differential survival from GEPIA (Hazards Ratio = 1.3, p-value = 0.038) (**Figure 4C**). The second was *UMODL1* with one missense mutation in its EGF-like calcium-binding (EGF_CA) domain, present in 29 patients (**Figure 4E**) as part of a significant mutation cluster with p-value = 1.11^e-19^ (**Figure 4F**) and yet again, a significant differential survival from GEPIA (Hazards Ratio = 0.74, p-value = 0.029) (**Figure 4B**).

### Mutations in *IRAK1* Lead to Structural Changes Impacting Stability and Binding of an Inhibitor

We chose *IRAK1* for further analysis based on its function as a modulator of the innate immune system and its association with survival. It is known that cancer cells escape the immune system due to faulty signalling.

**Figure 5:**
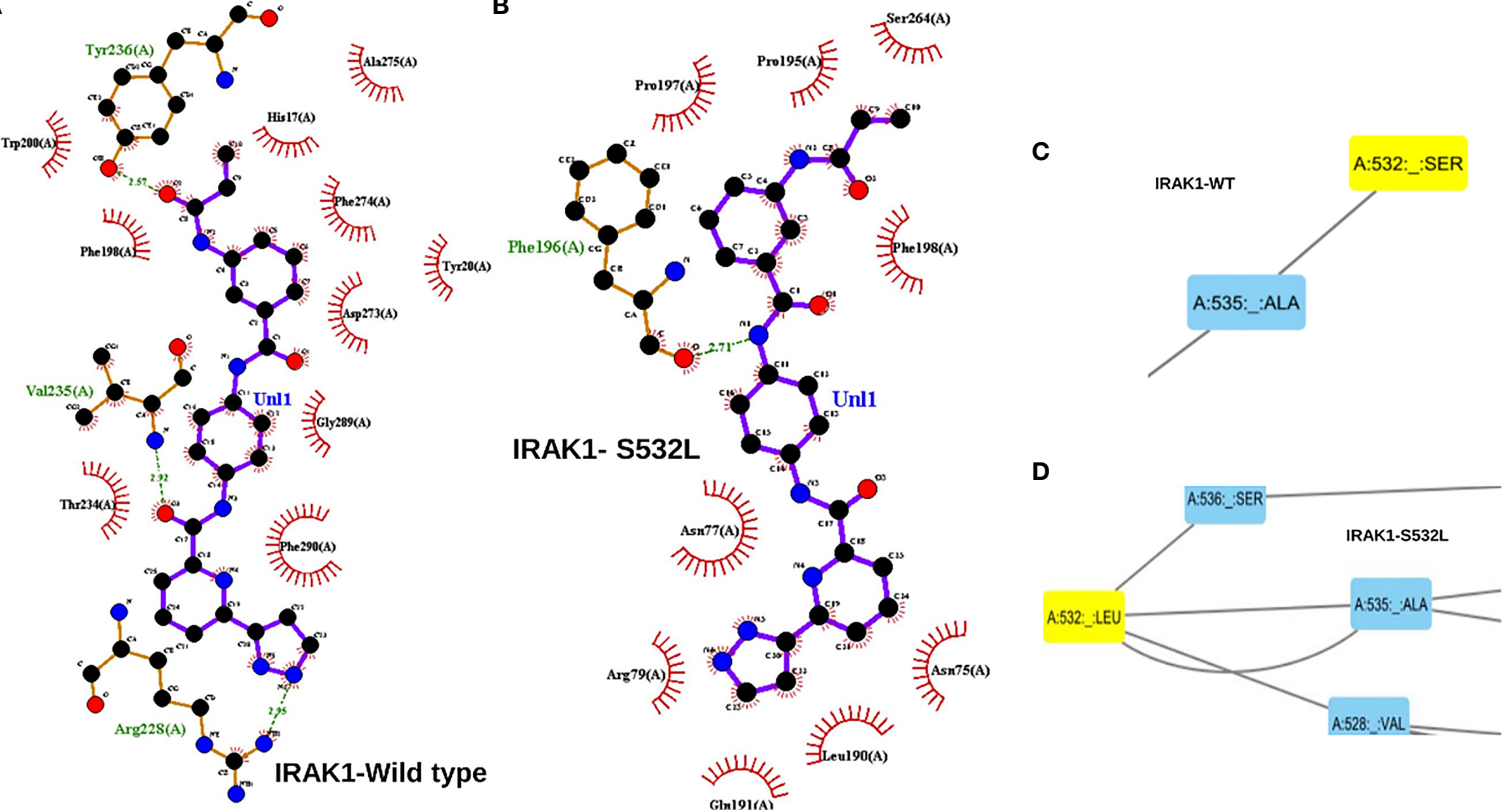
**(A)** A ligplot interaction image showing the interaction of JH-X-119-01 an IRAK1 inhibitor interacting with 3 residues of the wild type IRAK1 molecule. **(B)** Ligplot interaction image showing the interaction of JH-X-119-01 with just one residue of the S532L mutant IRAK1 molecule. The interactions in a and b have been marked in red circles. **(C)** A cytoscape screen shot showing the interaction of just two residues within the IRAK1 wildtype molecule. **(D)** Cytoscape screen shot showing increased interaction of 4 residues within the S532L mutant IRAK1 molecule.

The *IRAK1* protein structure and its two mutants S532L and F196S were modeled using the 712-residue sequence from UniProt (UniProt ID: P51617) on the Robetta web server. The modeled structure was then energy minimized, validated and the effects of the two mutations on stability and Gibbs free energy were analyzed. The energy of the structure modeled was found to decrease drastically upon energy minimization, from -22736.7 to -33353.3 for wildtype, from -22663.424 to -33286.121 for S53L and from -23651.352 to -33606.613 for F196S, indicating better structures for all the three (**Table 2**). The overall quality factor of the three structures, as predicted by SAVES web server was above 94, with the Wild structure having a factor of 96.1207 and S532L and F196S structures having a quality factor of 95.265 and 94.1176 respectively. In the case of S532L, the stability of IRAK1 structure increased up to a ddG value of 1.53 and decreased to a value of -0.52 for the F196S mutant as shown by Site Directed Mutator (SDM). Similar results were obtained using I-Mutant 2.0 (**Table 3**). JH-X-119-01, an inhibitor of IRAK1 (36) was docked on to the wild type *IRAK1* structure as well as the S532L and F196S mutant structures to study the change in ligand binding upon mutation. The active site of *IRAK1* (Serine/Threonine Protein Kinase) corresponds to residues 336-348. The binding site of *IRAK1*(Protein Kinase, ATP Binding Site) corresponds to residues 218-239. JH-X-119-01 interacts with Tyr236, Val235, and Arg228 on the wild type structure with a binding energy of -6.66 (**Figure 5A**, **Table 4**) and residues Arg232 and Tyr236 on the F196S mutant structure, with a slightly reduced binding energy of -5.46 (**Supplementary Figure S3A**, **Table 4**). The ligand did not have any favorable interactions with active site or binding site residues in the S532L mutant structure (**Table 4**). Ser532 on the wild type structure interacts with residue Ala535 only (**Figure 5C**) but Leu532 on the mutant type structure interacts with three residues (Val528, Ser536 and Ala535) (**Figure 5D**). This increase in residue interaction could explain the increase in stability upon mutation. Phe196 on the wild type structure interacts with three residues (Pro13, His17 and Phe18) but Ser196 on the mutant structure interacts with only one residue (Tyr20). This loss of two interactions could explain the decrease in stability upon mutation (**Supplementary Figures S3B, C**).

**Table 2:** Change in structure energy upon minimization.

Molecule Type	Structure Energy before minimization (in KJ/mol)	Structure Energy after minimization (in KJ/mol)
Wild	-22736.748	-33353.344
S532L	-22663.424	-33286.121
F196S	-23651.352	-33606.613

**Table 3:** ddG values for S532L and F196S mutants on SDM and I-Mutant 2.0.

Mutation	S532L	F196S
**SDM**	1.53	-0.52
**I-Mutant 2.0**	1.02	-1.62

**Table 4:** Docking energies and ligand-residue interactions.

Mutation Type	IRAK1 residues JH-X-119-01 interacts with	Binding Energy
**Wild**	Arg228	-6.66
	Val235	
	Tyr236	
**F196S**	Thr141	-5.46
	Arg232	
	Tyr236	
**S532L**	None of the docking conformations interacted with the active site or the binding site	

### Multivariate Prediction of Prognostic Markers Based on Survival Trends

**Figure 6:**
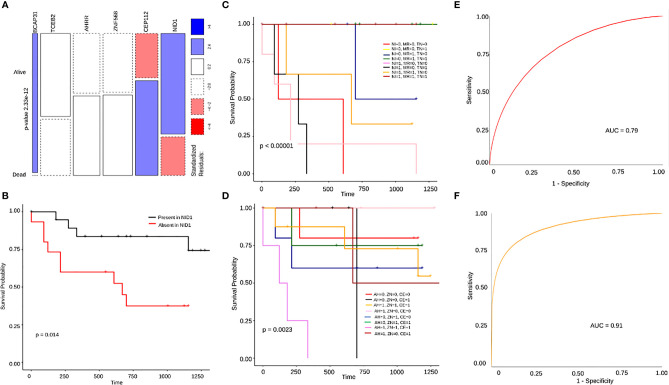
**(A)** A mosaic plot for chi square test showing significant difference between the number of alive and dead patients harboring mutations in BCAP31, TCEB2, AHRR, ZNF568, CEP112 and NID1. **(B)** A Kaplan Meier plot showing significant difference in survival probability between patients with and without NID1 mutation. **(C, D)** Are differential survival plots for the 3 dead and 3 alive gene signatures respectively. In **(C)** AH stands for AHRR, ZN stands for ZNF568 and CE stands for CEP112 where the difference in survival probabilities is much more significant with p=0.0023. In **(D)** NI stands for NID1, MR stands for MRPL23, and TN stands for TNS2, and the difference in survival between presence of mutations in different combinations of genes is significant with p<0.0001. **(E)** This is an ROC curve representing the 3 gene dead signature model with a high accuracy of 79% (AUC=0.79). **(F)** An ROC curve representing the 3 gene alive signature model with an even higher accuracy of 91% (AUC=0.91).

To identify survival associated markers, we used two approaches: we had the survival details of 37/51 samples, of which 23 were alive and 14 dead. To start with, we defined cut-off percentages for mutations to be considered as alive or dead signatures. We considered a particular variant as an alive signature only if the variant in a gene was present in at least 70% of the alive patients (~16/23) and present in utmost 40% (~6/14) or 50% (~7/14) of the dead patients. Similarly, for a variant to be considered a dead signature, we stated that it should be present in at least 60% of the dead patients (~10/14) and utmost 40% (~9/23) to 50% (~12/23) of the alive patients. Additionally, we also selected genes having mutations exclusively in alive or dead patients. By following these criteria, we shortlisted 17 genes for the alive signature and 19 genes for the dead signature (**Supplementary Table 1**). To screen these 36 genes further, we again referred to the HNSC differential survival dataset, based on the transcriptome, from the GEPIA database which resulted in 6 genes (**Supplementary Figure S4**). For alive signatures, we identified mutations in 3 genes, *BCAP31, TCEB2* and *NID1*. For dead signatures, we identified missense mutations in 3 genes, *AHRR, ZNF568* and *CEP112*. On performing a Chi-squared comparison test, there was a significant difference found between the alive and dead percentages of individuals for these 6 genes (**Figure 6A**). To check for differential survival concerning these 6 genes, Kaplan Meier plots were generated. Out of the 6 genes, *NID1* was the only gene that showed a significant differential survival between patients with mutations present in *NID1* and those with no mutation (p-value=0.014) (**Figure 6B**). The rest of the 5 genes, individually, did not show any significant differential survival with respect to presence or absence of mutations (**Supplementary Figures S5A–E**), though *BCAP31* showed borderline significance in its Kaplan-Meier plot (p-value=0.093) (**Supplementary Figure S5A**), and a significant difference in survival on clubbing it with *TP53* a known tumor suppressor (p=0.031) (**Supplementary Figure S5F**). Next, on clubbing the 3 genes alive and the 3 dead signature mutation data separately, we find a significant difference in survival (p-value=0.0048) for the dead signature as compared to the alive signature (p-value = 0.073) (**Supplementary Figures S5G**, **Figure 6C**). Additionally on performing a Multivariate Cox Proportional Hazards analysis of all the Clinical parameters clubbed with all the genes, it was observed that treatment group variables have a significant influence on patients’ risk of death (HR=18.7, p-value=0.044) (**Supplementary Figure S5H**) and in cases where clinical properties were clubbed with the individual genes one by one, only *TCEB2* and Treatment group variables, showed a significant influence on patient’s probability of survival (HR=0.013, p-value=0.021) and their risk of death (HR=17.003, p-value=0.002), respectively (**Supplementary Figure S5I**). Finally, we looked for the survival probabilities of these 6 genes in the HNSCC WEX data from the TCGA database and none of them showed a significant difference in survival. On analyzing the initial 36 genes again, independent of the GEPIA transcriptome data, we found significant differences in survival trends of 6 genes with an alive signature, *MRPL23, TNS2, SPECC1*, *TBP* and *PLXNA3*, and 2 genes, *MARCH10* and *COL4A6* as dead signature. The second approach was LASSO regression detailed below.

### Lasso Regression Models for Prediction of Prognostic Markers

**Figure 7:**
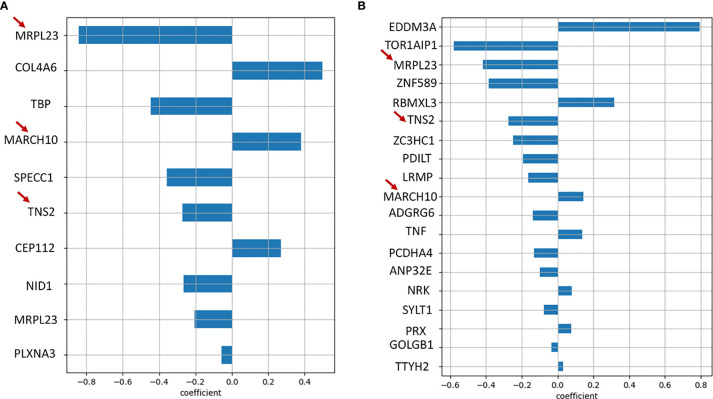
**(A)** Bar graph showing genes obtained as significant prognostic markers from Lasso regression algorithm of the initial 36 alive/dead genes. **(B)** Bar graph showing genes obtained as significant prognostic markers from Lasso regression algorithm of all genes with missense mutations, run using 100 random alpha values and 10000 iterations. It is interesting to note that MRPL23, TNS2 and MARCH10, marked with red arrows, are common to both the results.

In order to additionally screen for prognostic genes, we used LASSO regression to perform reduction analysis on two sets of genes; 1) The set of 36 genes obtained by applying our own cut-off criteria for dead and alive signatures, 2) Genes with only missense mutations present in at least 4 patient samples. The datasets were analyzed using python’s Scikit-learn module. The data was processed using 100 random penalty (alpha) values for 10000 iterations to obtain the best alpha value after 5-fold cross-validation. The best alpha values for both the datasets were 0.09 and.0817, respectively. On obtaining the best alpha value, we further obtained the coefficients for that particular alpha value which determine the significance of the associated genes. The genes with coefficients of 0 were eliminated. From the first dataset, we obtained *MRPL23* (-0.85), *COL4A6* (0.45), *TBP* (-0.45), *MARCH10* (0.38), *SPECC1* (-0.35), *TNS2* (-0.3) *CEP112* (0.28), and *NID1* (-0.3) as the genes with significant variants (**Figure 7A**). The same signatures were obtained from the second dataset: *MRPL23*, *TNS2*, *NID1*, and *MARCH10* (**Figure 7B**). Individual Kaplan-Meier plots of these genes showed significant differences in survival as well (**Supplementary Figures S6A–C**). Taking the genes common to the LASSO regression results using the two datasets and our initial independent scrutiny of significant survival genes, we observe that *MARCH10, MRPL23*, *NID1* and *TNS2* are statistically robust genes for a prognostic model prediction. All the above results were confirmed using the R package glmnet. To check if *MRPL23, TNS2 and NID1* would act as better alive gene signatures, we combined the survival data of all three and the difference in survival was significant (p-value < 0.0001) (**Figure 6D**). Adding *MARCH10* to the existing dead signature (*AHRR, ZNF568 and TCEB2*), resulted in a significant 4 gene signature (p-value = 0.012) (**Supplementary Figure S6D**). To check the accuracy of both the models, we performed and Receiver Operating Curve analysis (ROC) for both of them and the 3 gene alive model ROC curve resulted in an accuracy of 91% (AUC=0.91) and the 4 gene dead model (including *MARCH10*) had an accuracy of close to 80% (AUC=0.79). These results confirm the presence of two robust and accurate survival models; a 3 gene alive signature; *NID1, MRPL3* and *TNS2* and a 4 gene dead signature, *AHRR, ZNF568, CEP112* and *MARCH10* (**Figures 6E, F**). On comparing results from models of three sets of iterations, i.e., 10000, 1000, and 100 we observe a recurring occurrence of 2 genes *EDDM3A*, a secretory protein, and *TOR1AIP1*, nuclear laminar protein involved in the mTOR pathway (**Figure 7B**). Interestingly, both the genes also showed significant differences in survival when plotting Kaplan Meier graphs individually (**Supplementary Figure S6E, F**).

### Identification of a Novel and Deleterious Variant in Cancer Gene Census in Indian HNSCC

**Figure 8:**
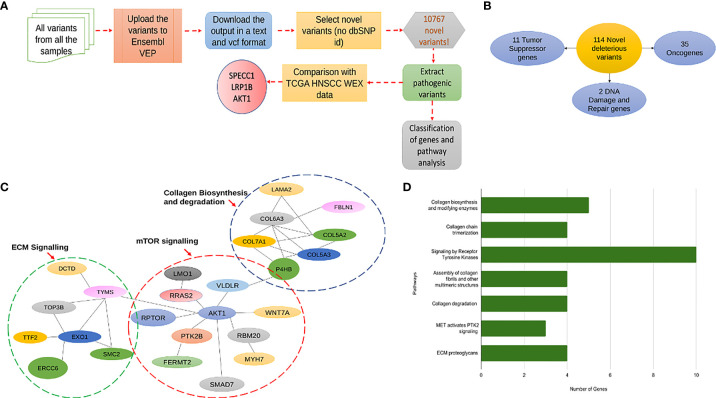
**(A)** A flowchart explaining the pipeline followed for prediction of novel deleterious variants from in-house data. **(B)** This diagram shows the classification of 114 novel deleterious variants into 11 Tumor suppressor genes, 35 oncogenes and 2 DNA damage and Repair genes. **(C)** This figure shows the distribution of the 114 novel deleterious variants into 3 significant pathway interaction networks, namely Collagen Biosynthesis and Degradation, mTOR signaling and ECM Signaling. **(D)** A bar graph showing all the significantly mutated pathways by the number of genes mutated with the most significant pathway being receptor tyrosine kinase signaling.

To check for the presence of novel variants in our data, from the merged vcf file of all the samples containing all the mutations, we separated out the first 5 columns namely the chromosome, the position of the mutation, reference allele, alternate allele, and the quality score and uploaded it onto the Ensembl Variant Effect Predictor (VEP). From the output file of VEP we filtered out the variants having neither a dbSNP -rs ID nor any COSMIC ID associated with it and termed these 10767 variants as novel variants. Further, we extracted pathogenic variants based on the “deleterious” factor from Sift and “probably_damaging” factor from the PolyPhen databases and obtained a list of unique genes associated with 114 novel variants of which 35 were oncogenes and 11 were tumor suppressors **(** **Figure 8A, B** **)**. On referring to the HNSCC whole exome data of these 114 genes from TCGA database we found that only 8 of them were a part of the Cancer Gene Census of which only 3 genes, *AKT1, LRP1B*, and *SPECC1* showed a significant difference in survival from the TCGA whole exome data associated with these genes (**Figure 8A**). Further, we looked into the association of the variants and the survival of the above-mentioned 3 genes in our data and found that *SPECC1* showed a significant difference in survival (p=0.035) (**Supplementary Figure 7A**). Surprisingly, the *SPECC1* variant that showed significant survival difference was an intron variant. We performed a network analysis of 114 genes associated with novel variants using the STRING and REACTOME database. Interestingly, the pathways with variants belonged to Collagen biosynthesis and degradation, mTOR signalling, and ECM signalling pathways (**Figure 8C, D**, **Supplementary Figure 7B**). The gene *AKT1*, a known oncogene, had the most significant number of interactions, interconnecting all the three major clusters observed.

### Copy Number Analysis

**Figure 9:**
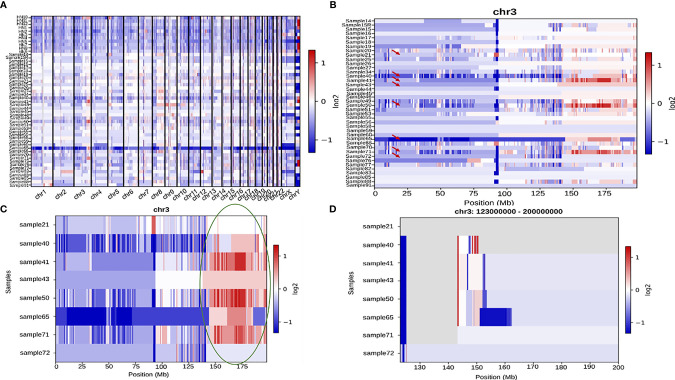
**(A)** A comprehensive heatmap showing copy number variation in all the samples across all the chromosomes. **(B)** Heatmap showing copy number variation across the entire length of chr3 in all the samples. Samples 21, 40, 41, 43, 50, 65, 71, and 72 are indicated by a red arrow since they show the most significant amount of variation amongst all the 41 samples. **(C)** A heatmap representing the above-mentioned subset of samples with a high amount of variation towards the end of chromosome 3. The last ~70 Mb (150Mb-200Mb) have been highlighted with a green oval which have been zoomed into in **(D)**.

CNVkit was used to perform CNV analysis of all the samples. Varying patterns of copy number gains and losses were seen in all the chromosomes across all the samples (**Figure 9A**). Based on initial results, 10 samples were excluded from further analysis as their patterns were collectively distinct from the rest 41. The remaining 41 samples chr3, chr7, chr8, chr17, chr19, and chrX (**Figure 9C**) showed distinct variations in copy number across their chromosome lengths. On continuing with chr3, which had the most striking number of variations, it was seen that within the chromosome, samples 21, 40, 41, 43, 50, 65, 71, and 72 displayed the most significant difference in copy number gain and loss between extreme ends of the chromosome (**Figures 9B, C**). Further, the last 70 Mb region of the chromosome in these 8 samples was analyzed and it was seen that *ZBTB38, ATP1B3, GK5, ZIC4, AGTR1, GYG1*, and *SERP1* genes showed a significant gain in Copy number (**Figure 9D**).

## Discussion

One of the main objectives of our study was to identify prognostic signatures linked to survival prediction in the Indian HNSCC cohort. In HNSCC, mutations in a known set of tumor suppressors and oncogenes, namely TP53, CCND1, NOTCH1, PIK3CA, MYC, CDKN2A, PTEN, and FBXW7, have been reported, but most of them are not associated with survival (4, 44). Several studies have reported a correlation of survival with specific signatures using either exome, or transcriptome, or small RNA signature (13, 45, 46). Several predictive models using machine learning algorithms such as random forest and lasso- cox regression have been developed to identify genes associated with treatment outcomes, survival, and prognosis of head and neck cancers (47, 48). There are no studies from India correlating survival with gene signatures. We have utilized LASSO-COX and developed a new method using integrated variant signature and gene expression to identify survival-associated genes. The variants identified from exome-see segregated the cancer of the buccal cavity from the tongue and alveolus. Previous studies from the Indian subpopulation on oral cancer and oesophageal cancer have identified mutations specific to the population (16, 49). We have identified novel mutations in AKT1, LRP1B, and SPECC1. Network analysis using all the novel variants identified Collagen biosynthesis and degradation, mTOR signalling, and ECM signalling pathways.

Preliminary variant analysis revealed that of the genes known to be mutated in HNSCC patients, *TP53 and TP63* were the ones with the highest frequency of mutation. Both these tumor suppressors are known to be mutated in HNSCC and loss of expression of the same has been linked to cancer progression while *TP63* is known to promote survival in HNSCC patients (4, 50, 51). Most of the other proto-oncogenes like *NOTCH1, FGFR1, EGFR, CCND1* or tumor suppressors like *CDKN2A, ARID2 and MLH1*, a mismatch repair gene, that are known to be frequently mutated in oral or head and neck squamous cell carcinomas in general were seen to have less than or equal to 20% mutation frequency. The frequency of these proteins being lower than usual, suggests heterogeneity especially in the Indian cohort.

On performing a tumor site-based signature analysis we came across several interesting results. To start with, *APC*, Adenomatous Polyposis Coli, a tumor suppressor in the WNT pathways, previously seen to have very low mutation rates in HNSCC was seen to have a structural interaction variant in 18 out of the 19 patients categorized. This suggests that the mutation was an inactivating mutation, contributing to the progression of HNC (52). Next, *GPC4*, Glypican 4, a known regulator of WNT signaling, known to be downregulated in breast cancer and ovarian cancer, and upregulated in colorectal cancer was seen to be mutated in 50% of the patients (52–54). The other genes with approximately 50% mutation frequency *WNT16, PARP1 and ATF4*. *PARP1* is known to have high expression levels in oral cancer and hence a more than 30% mutation frequency in our data, suggests an activating mutation in all tumor sites (55). The role of WNT16, a part of the canonical WNT signaling pathway family of genes, in cancer progression remains unknown, although expression of WNT16 is downregulated in Basal cell carcinoma (56).

Alveolar signatures were associated with mutations in cell cycle regulators such as *PRKCG, WEE1 and RBL2*. *PRKCG and WEE1*, when upregulated, are known to be good prognostic markers in Glioblastoma, while high mRNA levels of RBL2 are known to be associated with HPV+ head and neck tumors (57). Oncogenes like *FGFR1, HRAS, NRAS*, and a tumor suppressor CDKN2A are known to have mutations in HNSCC (4). Tumor suppressor *SMAD4*, involved in the EMT pathway was seen to have a stop gained mutation. It has been reported earlier that a somatic LOH mutation was present in a high frequency of lymph node metastatic tumors in HNSCC (58). The Buccal cavity signatures revealed a mutation in the cell cycle associated gene TFDP1, specific to the recurrent buccal sample. *TFDP1* amplification has been associated with lung cancer in a previous study and has been stated as a potential oncogene. Its role in head and neck cancer is unknown (59). *CASP8* and *ATM* were the signatures present in buccal cavity. Mutation frequency of 34% has been observed in *CAPS8* gene which is also associated with reduced survival in hnscc patients (60). Mutations in *ATM*, which is a well characterized tumor suppressor has been associated with oral cancer, lung cancer and breast cancer (61–63). The presence of a missense variant in a buccal mucosa tumor patient indicates that the mutation might be an inactivating mutation. Apart from *TP53*, which has previously been identified as a driver gene in oral tongue squamous cell carcinoma (64), novel gene cluster specific to tongue cancer has been identified which needs validation in larger cohort..

We investigated several potential prognostic markers based on a correlation of mutations occurring in genes and their corresponding survival outcomes. A comparison with the transcriptome data from the GEPIA database resulted in an initial set of 6 genes, 3 genes as a survival signature and 3 as dead. The most important finding out of the 6 survival signatures is the third alive signature gene *NID1*. Apart from showing a significant difference in survival from GEPIA, it was the only one that showed a significant difference in survival with in-house exome data. Nidogen 1 is a protein that interacts with several components of the extracellular matrix and its overexpression is known to correlate with drug resistance in ovarian cancer and increased metastasis in women affected with Breast Cancer (65, 66). Also, from GEPIA, high *NID1* levels correspond to low survival probability, while patients with *NID1* mutation show higher probability in our dataset, which suggests that the mutation is an inactivating mutation. Since the none of the other individual genes showed any significant difference in survival, we were encouraged to go back to our initial list of 36 genes and additionally screen through all the missense mutations in the data. A lasso regression model was built using python and R and the two datasets (36 genes and all missense mutations) were screened in order to obtain significant prognostic markers. From the algorithms and the initial curation results, we obtained 3 common alive signatures genes, MRPL23, TNS2 and NID1, and four dead signatures, MARCH10, AHRR, ZNF568 and CEP112. The individual association of survival probabilities of MRPL23, TNS2, MARCH10 and NID1 was significant.

Interestingly combined association of dead signatures showed a significant difference in survival. *AHRR*, an Aryl hydrocarbon receptor repressor, a known tumor suppressor has been associated with smokers in lung cancer patients in an epigenetic manner (67, 68). A mutation in the gene contributing to a dead signature suggests that its tumor suppressor potential was inactivated. The second dead signature gene was *CEP112*, a centrosomal protein involved in cell division, known to play a key role in the maintenance of genomic stability in association with *BRCA1 (* 69) and *ZNF568*, with the most significant contribution to the dead gene signature with a particular mutation present in 12 dead and 10 alive patients, indicating *ZNF568* role in tumor suppression.

Two ROC models were built to assess the accuracy of the 3 gene additive alive and the 4 gene dead signatures and it was observed that the alive signature was more accurate with an AUC of 0.91 while the accuracy of the dead signature was 0.79. There have been cancer studies where prognostic ROC models have been greater than 0.65 but have rarely crossed 0.87, which suggests that an AUC of 0.91 represents a significant predictive model (70, 71). Combining two different methods and selecting genes based on functionality rather than just the top signatures gave better accuracy than alone any of the methods, suggesting the potential robustness of this alternative approach towards screening of prognostic markers.

From these different sets of results, it is noteworthy that the predictive survival signatures are quite different when one considers the only exome as compared to when it is considered in concert with transcriptome analysis. Since the focus was on missense mutations, the same analysis also revealed targets with clinical implications and survival. We are aware that the cohorts for transcriptome and exome are not the same, exome is from our in-house Indian cohort while transcriptome is from GEPIA, representing mostly Caucasian population, nevertheless, the expression pattern with some degree of difference, more or less would be similar.

None of the Survival signatures showed significant survival differences in the Caucasian HNSCC cohort, which suggests the presence of population specific prognostic markers. We also performed preliminary Copy number analysis and obtained a signature differentiating a set of samples from another. Correlations between the clinical data/survival parameters with the copy number results are being investigated.

From the driver gene analysis, we saw that the second mutation of *IRAK1*, S532L showed significant results. S532L *IRAK1* mutant depicted the greatest deviation from the wild type in Docking studies and Residue Interaction Studies. The inhibitor (JH-X-119-01) was unable to interact with any active/binding site residues on the S532L mutant while it formed hydrogen bonds with the ATP binding site residues of the wild type and F196S variant. S532L mutant residue had added interactions with two residues which could have played a role in blocking the binding of the ligand to the binding site of the S532L mutant structure. F196S mutant residue had a loss of two interactions but was able to provide the binding pocket for ligand binding similar to the wild type. Further residue interaction analysis with the range of active site and binding site residues may shed light on the deviation in inhibitor binding behavior of the S532L mutant from the Wild Type *IRAK1*. Hence S532L seems to have a greater effect on the structure and ligand binding and can be targeted for further studies.

The small sample size of patients in the study is a definite shortcoming, but the use of multiple statistically significant methods supporting the findings of our alternate screening method, also resulting in a druggable protein target, reflects on the potential robustness of our method but the signatures obtained in this study need to be validated with a large set of patient samples.

## Conclusion

Exome sequencing and analysis of 51 HNSCC samples identified tumor site-specific biomarkers and a recurrence signature. The combined LASSO-COX and exome-transcriptome analysis of mutational profiles with clinical data resulted in 4 dead and 3 alive gene signatures linked to survival. The three genes alive signature identified can predict survival of HNSCC patients with 91% accuracy. We also identified novel mutations and a druggable driver gene target IRAK1. However, our results need validation with a larger sample size.

## Data Availability Statement

The datasets presented in this study can be found in online repositories. The names of the repository/repositories and accession number(s) can be found below: BioProject PRJNA740146.

## Ethics Statement

The study has been approved by the Institutional Ethics Committee of IBAB and HCG (IBABIEC-03/PR02/010518). Informed consent was collected from all the patients participating in the study.

## Author Contributions

BC and SD conceived and coordinated the study. AS and VR, provided the samples. BC, SD, and RR collected the samples. PS and RR performed the exome library preparation of the FFPE samples. BC, SD, RR, PD, GA, AJ, and SJ designed and performed the experiments. BC, SD, PD, and GA interpreted the data and wrote the manuscript. All authors contributed to the article and approved the submitted version.

## Funding

We thank the Department of Information Technology, Biotechnology and Science & Technology, Govt. of Karnataka, India, and Department of Science and Technology (SR/FST/LSI-536/2012), India for infrastructure grant and the Bio-IT grant. SD is supported by Department of Biotechnology (Ref. no BT/PR13458/COE/34/33/2015 and BT/PR13616/GET/119/9/2015), Govt. of India, India.

## Conflict of Interest

Authors AS and VR are employed by HealthCare Global Enterprises Ltd.

The remaining authors declare that the research was conducted in the absence of any commercial or financial relationships that could be construed as a potential conflict of interest.

## Publisher’s Note

All claims expressed in this article are solely those of the authors and do not necessarily represent those of their affiliated organizations, or those of the publisher, the editors and the reviewers. Any product that may be evaluated in this article, or claim that may be made by its manufacturer, is not guaranteed or endorsed by the publisher.

## Supplementary material

Supplementary Figure S1 A waterfall plot showing site wise signatures mutations across patients with site of tumor originating from alveolus, buccal mucosa and tongue.

Supplementary Figure S2
**(A)** A K-M plot showing difference in survival probabilities between different stages of tumor. **(B)** A K-M plot showing significant difference in survival probabilities between different types of chemotherapy regimens.

Supplementary Figure S3
**(A)** A ligplot image showing the interaction of JH-X-119-01 with F196S mutant IRAK1 molecule, marked in red circles. **(B)** A cytoscape image showing internal IRAK1 wildtype interactions. **(C)** Cytoscape image showing a reduced number of interactions within the IRAK1 molecule.

Supplementary Figure S4 A panel of KM plots from GEPIA showing significant differences in survival probabilities of BCAP31, AHRR, ZNF568, TCEB2, CEP112 and NID1 based on high and low expression categories.

Supplementary Figure S5
**(A–E)** Showing K-M plots of BCAP31, AHRR, ZNF568, CEP112 and TCEB2 based on presence or absence of mutations. Note that the differences in survival probabilities are not significant. **(F)** A KM plot of BCAP31 and TP53 combined showing significant difference in survival based on presence or absence of mutations in both the genes. **(G)** An additive differential survival plot of the initial 3 alive gene signature, BCAP31, TCEB2 and NID1, showing a low significance value of 0.073. **(H)** A multivariate analysis forest plot showing significant influence of Treatment group of variables on patients’ risk of death. **(I)** A multivariate forest plot showing significant influence of Treatment group of variables on patients’ risk of death.

Supplementary Figure S6
**(A–C)** K-M plots showing significant difference in survival probabilities between absence and presence of mutations in 3 genes, MRPL23, MARCH10 and TNS2. **(D)** And additive differential plot of the 4 gene dead signature, AHRR, ZNF568, CEP112 and MARCH10, showing significant difference in survival probabilities. **(E)** A K-M plot showing significant difference in survival between two categories of EDDM3A gene based on presence or absence of mutations. **(F)** A K-M plot showing significant difference in survival between two categories of TOR1AIP1 gene based on presence or absence of mutations.

Supplementary Figure S7
**(A)** A K-M plot showing significant difference in survival between two categories of SPECC1 gene based on presence or absence of an intron variant. **(B)** An interaction network obtained from the STRING database, showing 3 significant pathways from the novel deleterious genes linked by AKT1.
