# Supplementary material for: Multidimensional Mutational Profiling of the Indian HNSCC Sub-Population Provides IRAK1, a Novel Driver Gene and Potential Druggable Target
Source: Front Oncol. 2021 Nov 2;11:723162. doi: 10.3389/fonc.2021.723162 (PMC8593415; doi:10.3389/fonc.2021.723162)
Supplement: Supplementary Figure S1 — A waterfall plot showing site wise signatures mutations across patients with site of tumor originating from alveolus, buccal mucosa and tongue. [file Presentation_1.pptx]

## Slide 1
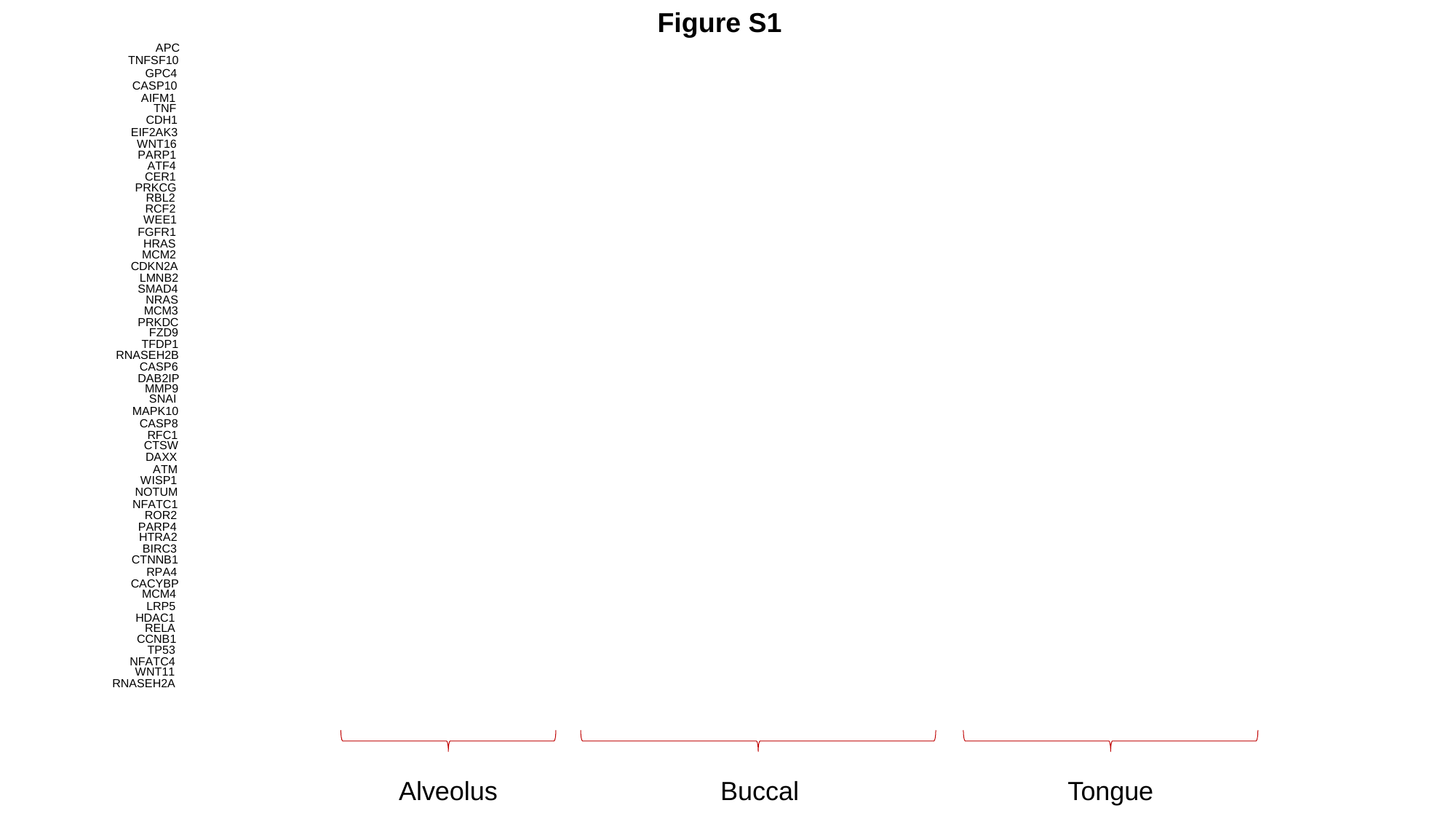

Figure S1

## Slide 2
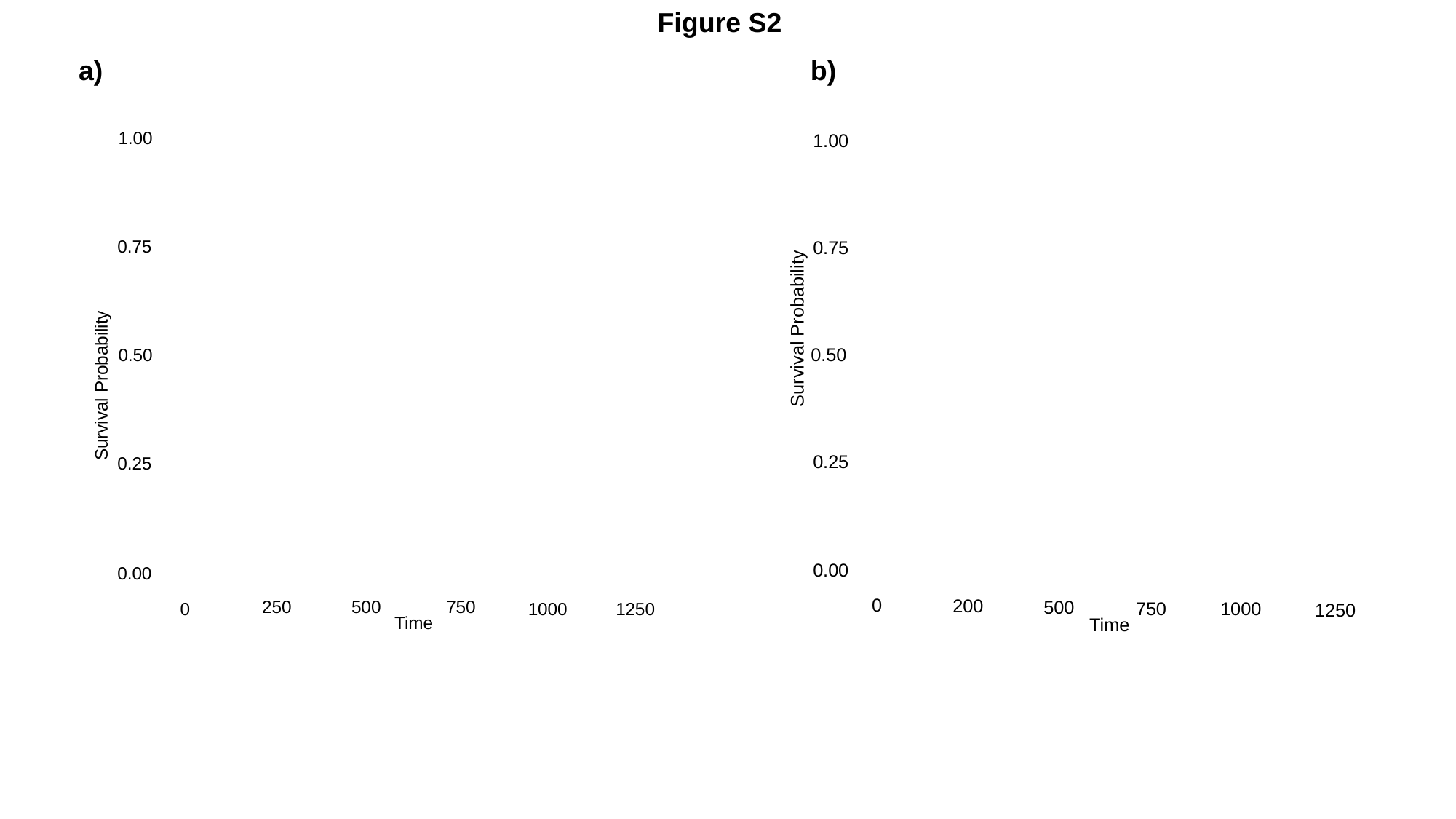

Figure S2
a)
b)

## Slide 3
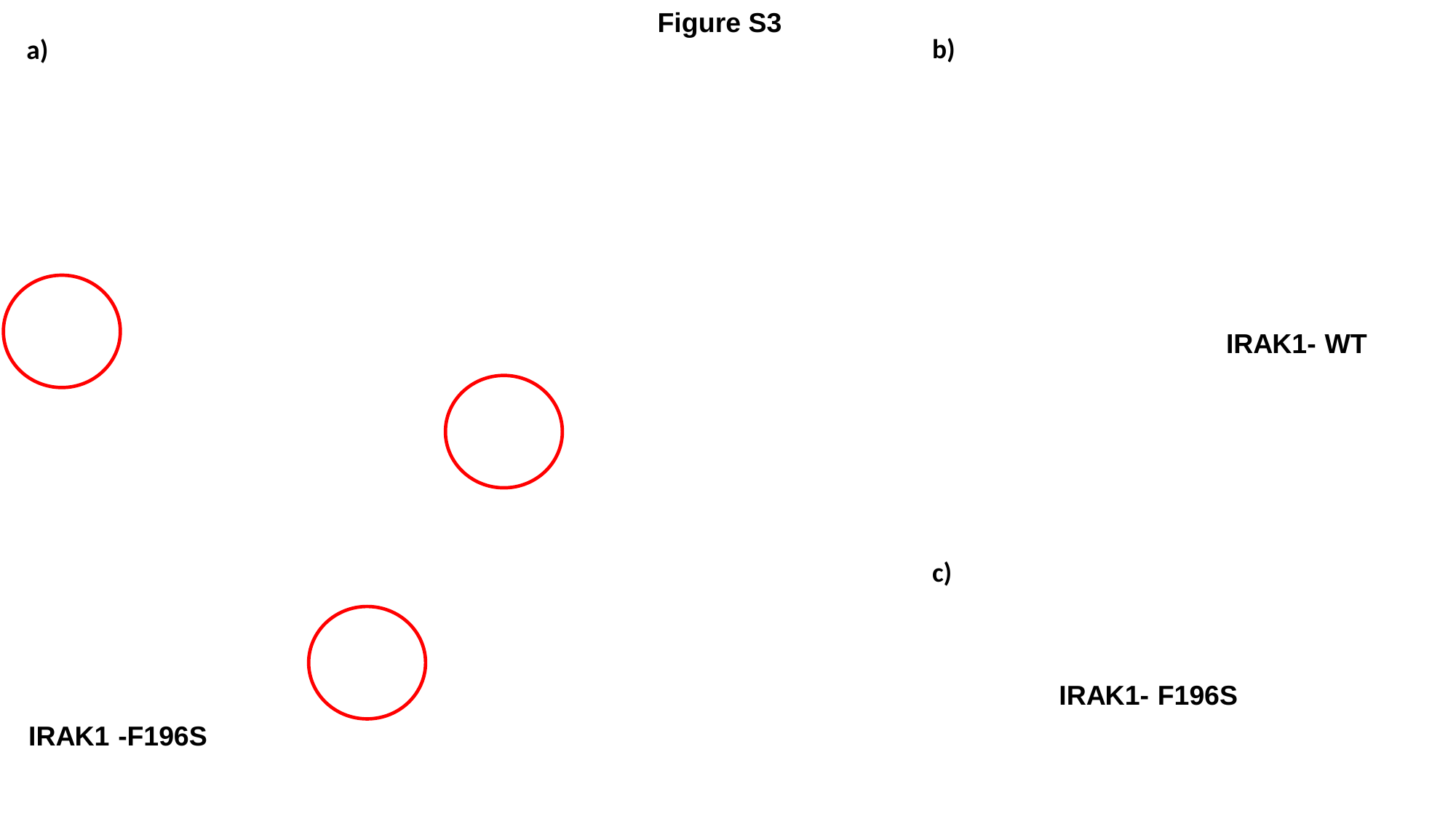

Figure S3
b)
a)
c)

## Slide 4
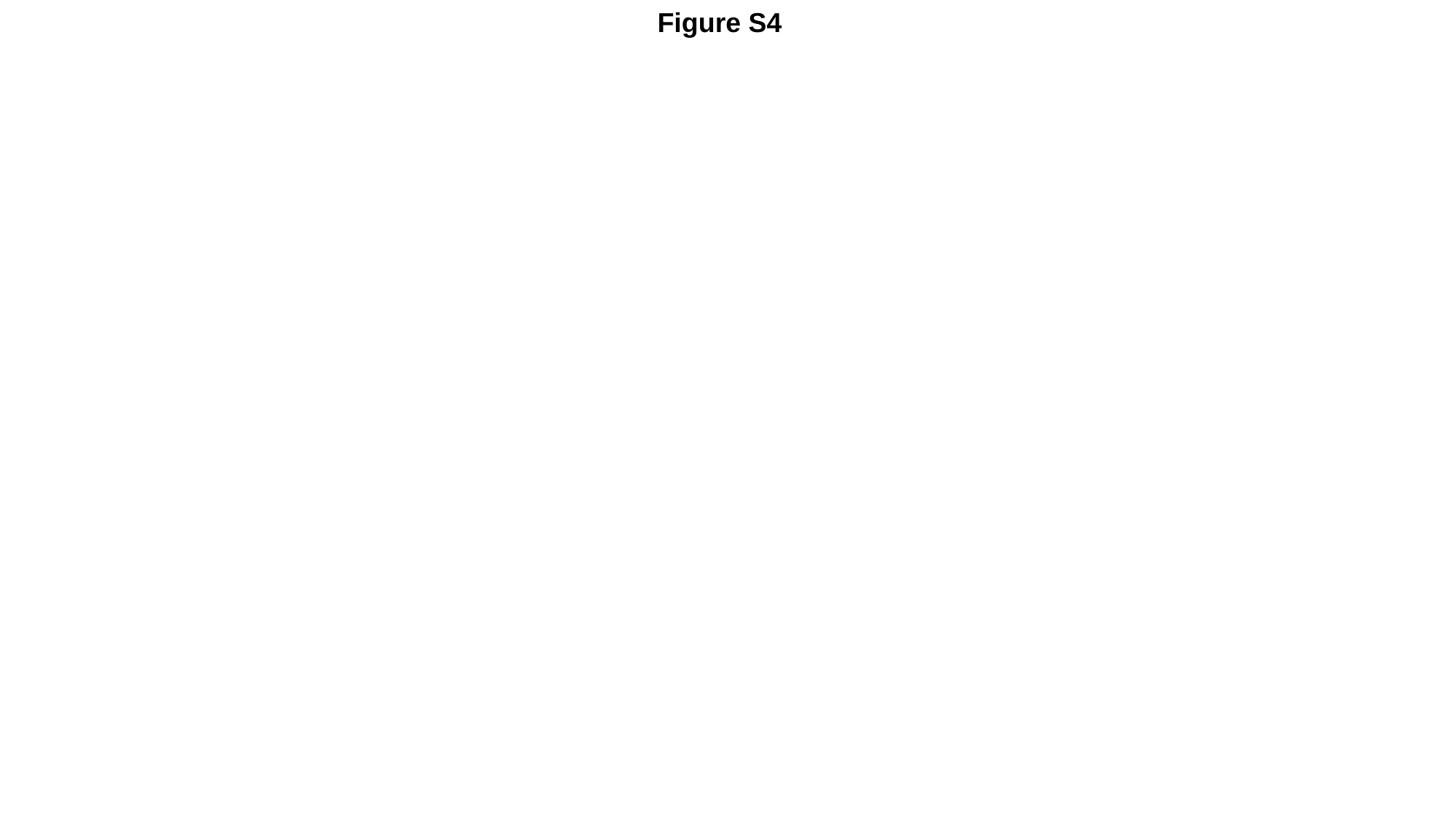

Figure S4

## Slide 5
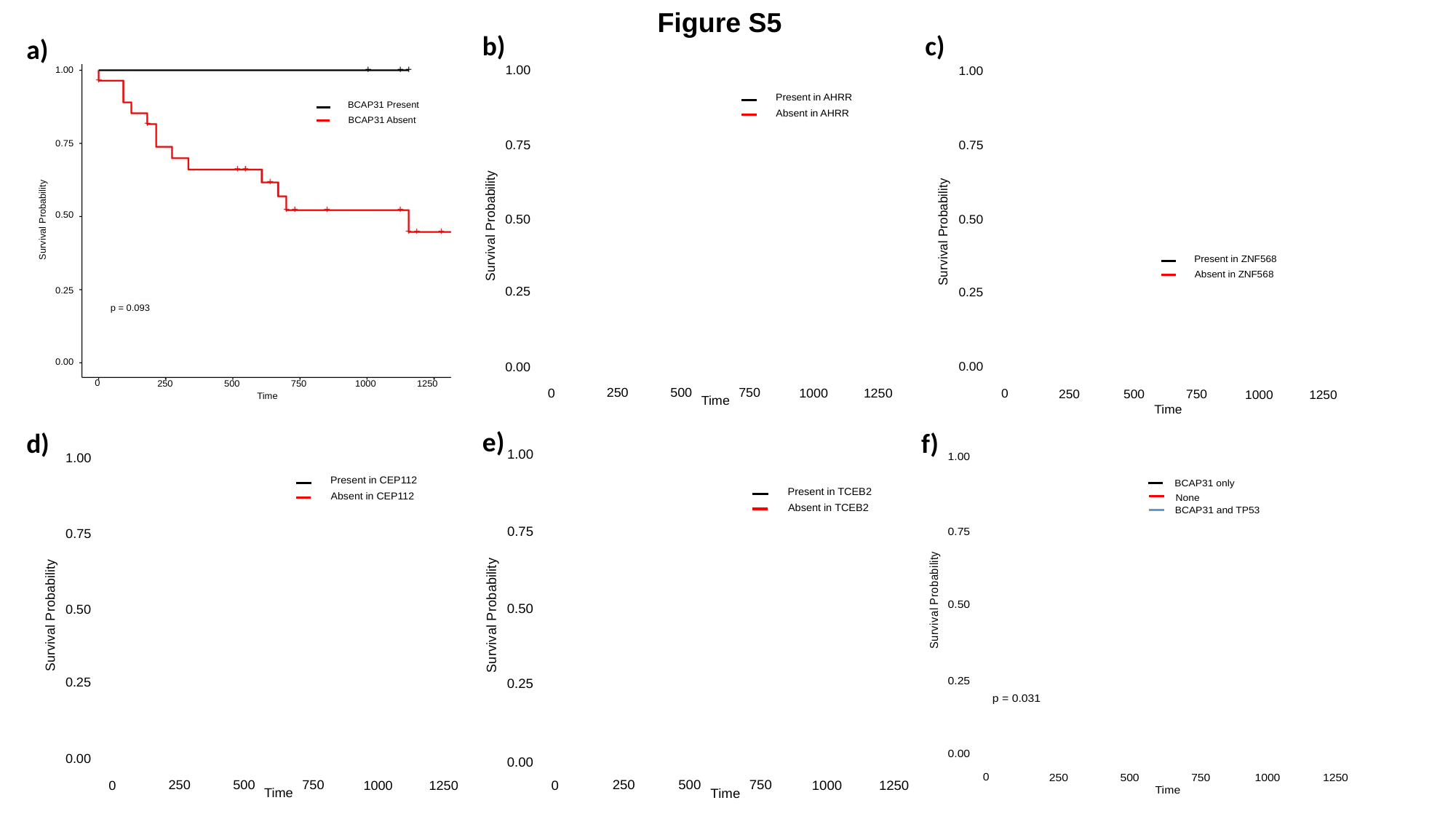

Figure S5
b)
c)
a)
e)
d)
f)

## Slide 6
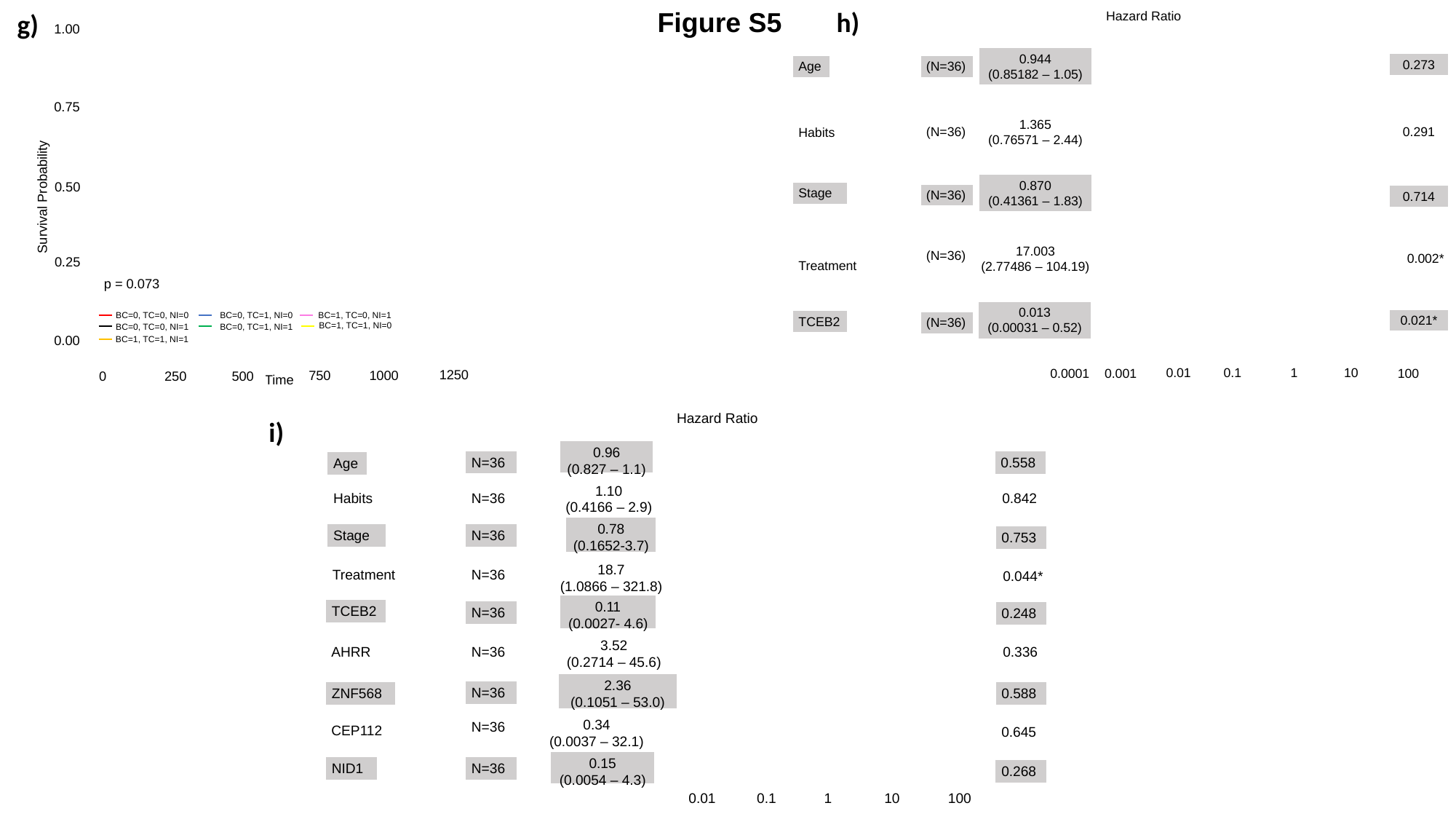

Figure S5
h)
g)
i)

## Slide 7
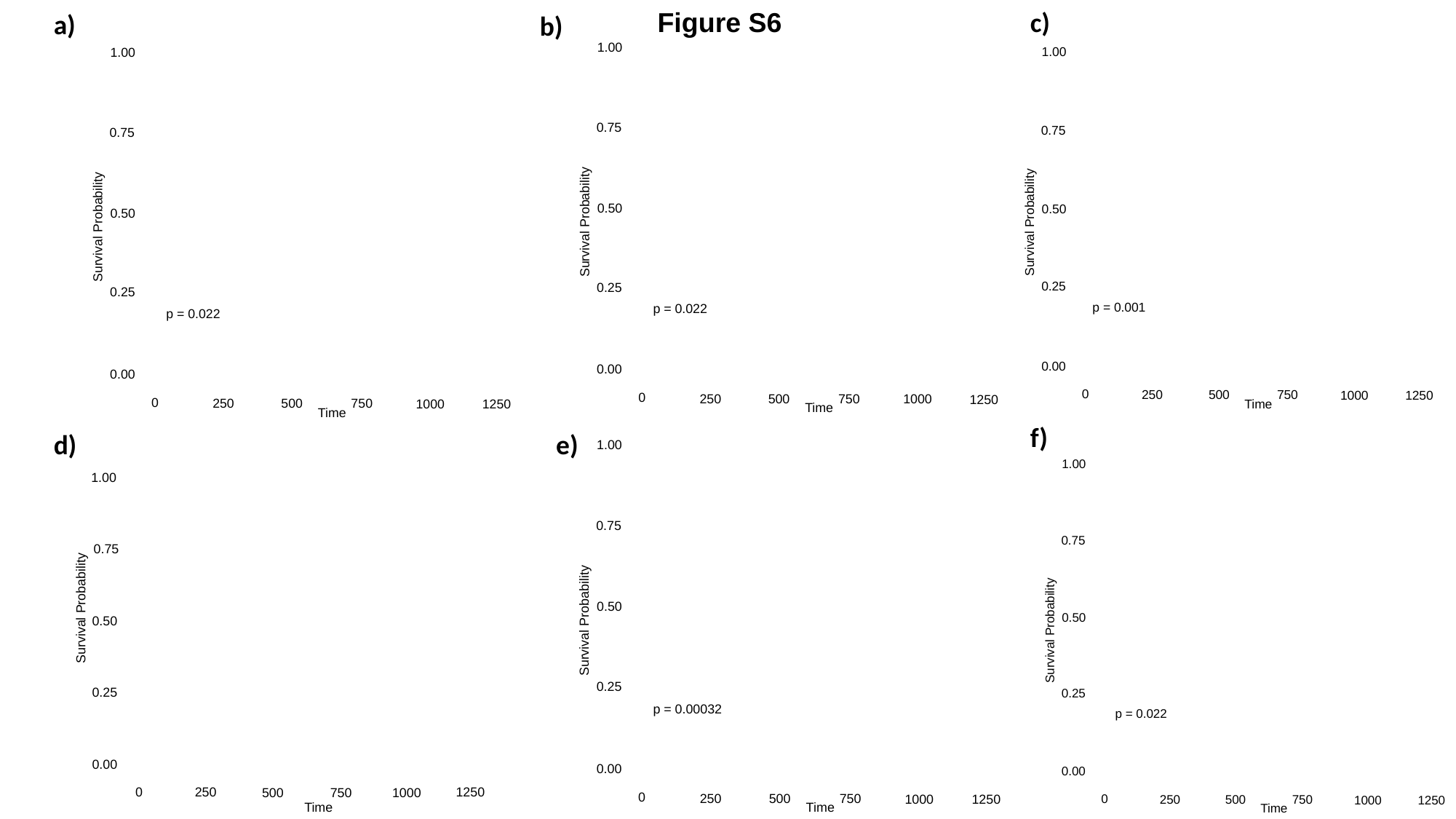

Figure S6
c)
a)
b)
f)
d)
e)

## Slide 8
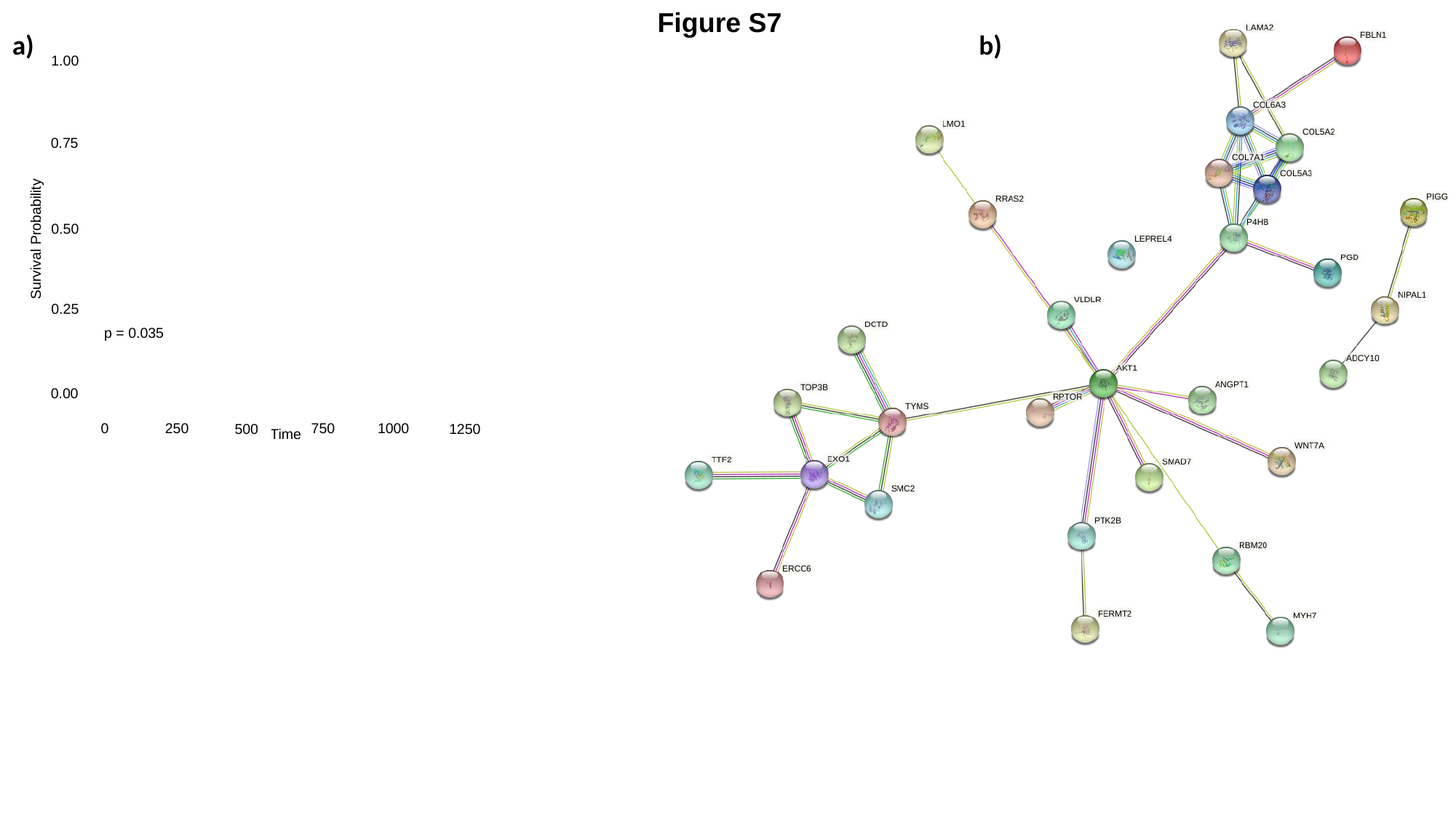

Figure S7
a)
b)
